# Analysis of immunogenetics interlaboratory comparisons’ success rates. External quality assurance system of the Spanish Society for Immunology GECLID-SEI

**DOI:** 10.3389/fgene.2024.1268728

**Published:** 2024-04-30

**Authors:** M. Carmen Martín

**Affiliations:** Centro de Hemoterapia y Hemodonación de Castilla y León, Valladolid, Spain

**Keywords:** human leukocyte antigen, immunogenetics, proficiency testing, molecular genetics, crossmatch, chimerism, killer inhibitory receptor, quality assurance

## Abstract

**Background:**

For many years, transplantation outcomes were uncertain and not hopeful, until histocompatibility testing spread. Common criteria for histocompatibility assays and communications’ improvement allowed an efficient organ sharing system. The possibility of organ exchanges is closely linked to the importance of interlaboratory comparisons for histocompatibility and immunogenetics methods. The external proficiency testing (EPT) systems are the most powerful quality assurance tools. They help achieve harmonization of analyses, set a standard of performance, and a common interpretation.

**Methods:**

The external quality assurance program for diagnostic immunology laboratories (Garantía Externa de Calidad para Laboratorios de Inmunología Diagnóstica, GECLID) program nowadays runs 13 external quality assurance (EQA) histocompatibility and immunogenetics schemes, with the first of them from 2011 to date: serological and molecular: low- and high-resolution human leukocyte antigen (HLA), human platelet antigen (HPA), and killer inhibitory receptor (KIR) typing(HLA-B*27, HLA-B*57:01, and coeliac disease-related HLA), cell-dependent cytotoxicity (CDC) and flow cytometry (FC) crossmatches, anti-HLA and anti-HPA antibodies, and chimerism.

**Results:**

A total of 85 laboratories participated in this subprogram in the last 12 years reporting over 1.69 M results: 1.46 M for anti-HLA and anti-HPA antibodies, 203.810 molecular typing data (HLA, HPA, and KIR genes), 2.372 for chimerism analyses, and 39.352 for crossmatches. Based on the European Federation for Immunogenetics (EFI) standards for EPT providers, the mean success rates ranged from 99.2% for molecular typing schemes and antibodies and 94.8% for chimerism, was 96.7% regarding crossmatches, and was 98.9% in serological typing. In 2022, 61.3% of the participating laboratories successfully passed every HLA EQA scheme, although 87.9% annual reports were satisfactory. Most penalties were due to nomenclature errors or misreporting of the risk associated to HLA and disease.

**Conclusion:**

This EQA confirms the reliability of HLA and immunogenetics assays in routine care. There is little heterogeneity of results of different assays used by participating laboratories, even when in-house assays are used. Reliability of test results is reasonably granted.

## 1 Introduction

The Spanish Society for Immunology founded in 1975 is a professional non-profit organization in Spain and is dedicated to promote and support excellence in research, scholarship, and clinical practice in immunology. The external quality assurance (EQA) program for diagnostic immunology laboratories (Garantía Externa de Calidad para Laboratorios de Inmunología Diagnóstica, GECLID) program was first run in 2011 (Martín et al., 2011), and more than 130 laboratories all around the world take their interlaboratory comparisons for proficiency testing nowadays.

The European Federation for Immunogenetics (EFI) in their standards for external proficiency testing (EPT) provides the criteria required for organizations providing EPT services to conform to the accreditation procedures of the EFI (European Federation for Immunogenetics, 2021). The standard comprises recommendations on the organization of an EQA scheme, the requirements for EQA test samples and their evaluation guidelines for an EQA scheme. They propose a scoring system and the mandatory contents of EQA reports, as well as information to participants. ISO 17043 (International Organization for Standardization, 2023) states the general interlaboratory comparison rules to ensure their quality, and ISO 15328 (International Organization for Standardization, 2022) gives advice on how to analyze reported data and calculate assigned values to parameters in EPT exercises.

Proficiency testing provides information on the accuracy of reported results, and subsequently on the clinical performance and on the correct interpretation of results. This is especially relevant regarding laboratory-developed or laboratory-modified tests (LDTs). The EU *in vitro* Diagnostic Device Regulation (European Parliament, 2017) requires appropriateness evaluation for LDTs. They can be evaluated by comparison with published biological variation estimations, and by participation in PT schemes or by comparison with their published results (Comins et al., 2023)

There are plenty of methods and variants for human leukocyte antigen (HLA) testing, but laboratories have to yield homogeneous results to grant patient’s safety. Methodological differences together with environmental, personal, or eventual issues can affect laboratories’ performance. Pre- and post-analytical issues should be noticed as well. The last aim of a clinical laboratory is to ascertain that their results are true in spite of all handicaps, and this is where EQA plays a key role.

EPT programs are committed to design useful schemes resembling routine work as much as possible. Samples should be representative of the variety of clinical ones; participants should perform their analysis as they usually do and report parameters following their practice. Once all results have been recorded, the assigned values are determined by consensus of participants and ratified by the steering committee. Global reports containing every single lab results (properly anonymized) together with private individual summaries are published. Appeals are as well evaluated by the steering committee.

In the present work, participation, success rates, and mistakes of the last 12 EPT years are described and analyzed to identify relevant factors that might influence error sources or changes in performance over time, within analysis families or by scheme.

## 2 Materials and methods

The GECLID program nowadays runs 13 external quality assurance (EQA) HLA and immunogenetics schemes (the first of them from 2011 to date): serological and molecular typing (low and high resolution HLA, human platelet antigen (HPA), killer inhibitory receptor (KIR), HLA-B*27, HLA-B*57:01 and coeliac disease-related HLA), anti-HLA and anti-HPA antibodies, crossmatches and chimerism (Table 1). Molecular typing schemes, antibodies, and crossmatch schemes are ISO 17043 accredited by our national accreditation body (ENAC, Entidad Nacional de Acreditación) from 2023 summer. Schemes are grouped in five analytical families: serology, molecular typing, antibodies, chimerism, and crossmatches.

**TABLE 1:** Interlaboratory comparisons provided by the GECLID program and short names.

Scheme	Short name	Analytical family
**Serological typing of HLA class I**	**SER**	**Serology**
**HLA-B27**	**B27**	**Molecular typing**
HLA*B57:01	**B57**	**Molecular typing**
**Coeliac disease–related HLA**	**CD**	**Molecular typing**
**Cytotoxicity crossmatch**	**XM**	**Crossmatch**
**Cytometry crossmatch**	**XMFC**	**Crossmatch**
**Detection and specificity of anti-HLA antibodies**	**ALO**	**Antibodies**
**Low-resolution HLA DNA typing**	**LOW**	**Molecular typing**
**High-resolution HLA DNA typing**	**HI**	**Molecular typing**
**Chimerism**	**CHM**	**Chimerism**
**KIR typing**	**KIR**	**Molecular typing**
**HPA typing**	**HPA**	**Molecular typing**
**Anti-HPA antibodies**	**AHPA**	**Antibodies**

Values are assigned according to EFI rules (European Federation for Immunogenetics, 2021). Success rates are calculated as the total number of error-free results over valid data (not inconclusive or unevaluable items).

The principal objective of the program is to assess the performance of participants by comparing results from the full range of analytical methods presently used in the immunology laboratories of histocompatibility and immunogenetics. Participation is not restricted by the method; laboratories should perform their protocols as they routinely do.

Eighty-five laboratories have participated in any scheme of the subprogram in the last 12 years (Table 2). All schemes but serological typing have increased their participation over time. Spanish laboratories are the most frequent ones, but Portuguese, Czech, Serbian, and Chilean ones have been participating to date as well. Laboratories from Israel and Kazakhstan have formerly participated.

**TABLE 2:** Groups of schemes by descending mean number of participants in the last year.

		2011	2012	2013	2014	2015	2016	2017	2018	2019	2020	2021	2022
Molecular typing	B27	25	25	24	29	29	33	38	37	37	40	41	47
	CD	—	17	20	24	26	33	37	39	42	42	44	45
	LOW	33	33	34	36	35	37	38	38	39	42	43	44
	B57	—	12	14	18	23	26	29	31	33	36	40	40
	HI	28	28	28	28	28	30	31	29	34	33	33	34
	KIR	—	—	—	—	—	16	21	22	23	22	22	25
	HPA	—	—	—	—	—	—	5	4	6	6	7	6
Crossmatch	XM	23	22	22	22	22	23	24	22	23	24	25	27
	XMFC	—	7	10	8	9	10	13	14	15	16	18	17
Antibodies	ALO	27	29	29	30	31	30	34	33	33	34	36	41
	AHPA	—	—	—	—	—	—	—	—	5	6	7	7
Chimerism	CHM	—	—	7	10	10	12	14	14	18	15	16	16
Serology	SER	18	18	16	16	12	11	12	11	9	9	6	5
	Total	40	43	45	50	54	58	64	64	64	65	71	75

Informed consents were obtained from any individuals included in this study. Samples within the histocompatibility GECLID subprogram are always of human origin, with minimal handling, so that they are as similar as possible to the usual practice of diagnostic laboratories. The methods employed in the preparation and distribution of samples have been shown to be suitable to ensure uniformity and stability in the conditions listed. Samples are peripheral blood (buffy coats) and sera. All the manipulation is performed under sterile conditions. Most of the samples included in this subprogram come from the Biobanco del Centro de Hemoterapia y Hemodonación de Castilla y León (Valladolid, Spain). Also, patients’ samples can be obtained from different blood banks and clinical services of the Spanish territory in accordance with current legislation on the subject. The GECLID program has the approval of both the Scientific Committee of the Biobank and the Valladolid Este Ethics Committee.

All samples are distributed in suitable packaging, in accordance with the International Air Transport Association (IATA) standard and are accompanied by documentation (pdf documents sent by e-mail for the sake of a better sustainability) with at least the sample number and lot, additives and/or preservatives included, and analytical tests expected to be carried out on each sample by participant laboratories. All samples included in the schemes have a documented traceability system: origin, serology, staff involved in handling and packaging, date of extraction and shipping, etc. GECLID-SEI keeps a part of each batch of samples for at least 1 year, so that laboratories can acquire on request extra volumes and can reanalyze them, if necessary.

Laboratories’ results can be reported exclusively by means of the web results forms available at https://www.geclid.es/ according to the guidelines in the HLA prospectus. The prospectuses from every EPT year are publicly available at the website. The analysis of frequencies and robust mean calculation from all results reported by the participants [an algorithm described in Annex C of ISO 13528 (International Organization for Standardization, 2022)] are performed to assign correct values to each parameter.

## 3 Results

Along these 12 years, 1.69 M results have been recorded, analyzed, and evaluated: 1.46 M for anti-HLA and anti-HPA antibodies, 203.810 molecular typing data (HLA, HPA, and KIR genes), 2.372 for chimerism analyses, and 39.352 for both cell-dependent cytotoxicity (CDC) and flow cytometry (FC) crossmatches (Table 3).

**TABLE 3:** Data reported by year and scheme.

	2011	2012	2013	2014	2015	2016	2017	2018	2019	2020	2021	2022	Total
ALO	14,681	66,200	68,425	69,220	68,699	74,893	73,101	130,260	170,496	187,605	210,847	329,698	1,464,125
LOW	3,161	3,659	3,683	3,482	4,002	3,257	5,508	6,636	7,980	6,840	7,980	6,660	62,848
HI	2,915	3,503	3,403	3,387	3,674	3,234	4,869	6,488	7,705	6,380	7,364	6,160	59,082
XM	1,973	1,112	1,078	1,133	1,077	1,168	1,838	3,382	3,780	3,612	4,368	2,352	26,873
KIR	0	0	0	0	2,383	0	3,541	4,016	4,200	3,538	4,500	3,833	26,011
XMFC	0	831	787	684	892	582	1,551	1,442	1,568	1,230	1,792	1,120	12,479
CD	0	756	690	445	914	345	1,665	1,408	1,600	1,170	1,760	1,095	11,848
SER	1,250	773	976	1,026	579	1,199	296	541	560	640	400	760	9,000
B57	0	942	320	200	846	170	1,050	797	1,240	775	1,540	724	8,604
B27	199	282	278	187	297	191	705	497	750	370	890	380	5,026
HPA	0	0	0	0	0	0	698	600	600	400	720	500	3,518
CHM	0	0	168	152	110	190	281	320	290	260	321	280	2,372
AHPA	0	0	0	0	0	0	0	0	166	175	139	240	720

Scheme design, that includes evaluation of results, is based on the EFI Standards for EPT providers (European Federation for Immunogenetics, 2019).

The mean success rates are above 99.0% for low-resolution HLA and KIR molecular typing, anti-HLA and anti-HPA antibodies detection and identification, and HLA-B*57:01 determination (Table 4). PCR-SSO (sequence-specific oligotyping) was the preferred method for HLA-B*57:01, coeliac disease–related HLA, low-resolution DNA HLA typing, KIR typing, and HPA typing, whereas PCR-SBT (sequencing-based typing) was the elected method for high-resolution HLA DNA typing until 2020, when it became the second elected choice. Next-generation sequencing (NGS) has been preferred from then to date. Sequence-specific primer PCR (PCR-SSP) alone or together with PCR-SSO was the second most used method for low-resolution KIR, HLA, and HPA typing. Real-time PCR (rtPCR) was the second most used molecular method for related disease HLA (B27, B*57:01, and coeliac disease-related HLA). The most frequent tests for antibodies were Luminex-based assays—single antigen regarding anti-HLA antibodies (Table 5).

**TABLE 4:** Success rates by year and group of schemes.

		2011 (%)	2012 (%)	2013 (%)	2014 (%)	2015 (%)	2016 (%)	2017 (%)	2018 (%)	2019 (%)	2020 (%)	2021 (%)	2022 (%)	Mean (%)
Molecular typing	HPA							100.0	100.0	99.0	99.5	100.0	100.0	99.8
	KIR						98.9	99.4	99.3	99.7	99.8	99.7	99.9	99.5
	LOW	99.0	98.9	99.0	99.5	99.2	99.3	99.2	99.8	99.8	99.6	99.8	99.9	99.4
	B57		98.8	99.5	99.7	100.0	98.1	100.0	98.8	99.0	99.9	99.2	99.8	99.4
	HI	99.0	99.0	99.1	99.5	99.3	99.6	98.7	99.1	99.1	99.0	98.0	98.6	99.0
	B27	99.5	99.5	95.7	99.3	98.9	99.3	98.7	98.4	99.8	99.5	99.2	99.9	99.0
	CD		96.8	99.1	99.3	99.3	95.3	98.9	97.8	98.1	98.8	98.4	99.6	98.3
Antibodies	AHPA									100.0	98.3	99.3	100.0	99.4
	ALO	97.7	98.7	99.1	98.7	98.3	98.6	99.8	99.4	99.3	99.5	99.3	99.7	99.0
Serology	SER	96.2	96.0	98.9	99.6	99.4	99.8	99.7	99.5	99.3	99.3	99.3	100.0	98.9
Crossmatch	XM	96.2	97.2	97.4	97.5	97.3	95.0	99.2	99.3	98.3	99.6	96.6	99.2	97.7
	XMFC		94.9	94.7	97.3	95.1	94.1	95.2	94.5	95.4	97.1	95.3	97.8	95.6
Chimerism	CHM			88.2	92.1	97.0	95.8	96.4	94.6	93.8	96.6	95.7	97.8	94.8

**TABLE 5:** Most performed method by year and group of schemes.

		2011	2012	2013	2014	2015	2016	2017	2018	2019	2020	2021	2022
Molecular typing	HPA	—	—	—	—	—	—	SSO	SSP	SSP	SSO	SSO	SSO
								40.00%	50.00%	66.67%	50.00%	57.14%	50.00%
	KIR	—	—	—	—	—	SSO	SSO	SSO	SSO	SSO	SSO	SSO
							91.67%	75.00%	73.68%	60.00%	70.00%	73.68%	59.09%
	LOW	SSO	SSO	SSO	SSO	SSO	SSO	SSO	SSO	SSO	SSO	SSO	SSO
		66.67%	64.70%	66.67%	72.22%	77.14%	70.59	67.57	71.05	72.5	67.5	70.45	70.73
	B57	—	SSO	SSO	SSO	SSO	SSO	SSO	SSO	SSO	SSO	SSO	SSO
			45.45%	46.15%	52.94%	47.83	41.64%	40.91%	48.28%	48.28%	51.61%	50.00%	54.55%
	HI	SBT	SBT	SBT	SBT	SBT	SBT	SBT	SBT	SBT	NGS	NGS	NGS
		33.33%	48.14%	46.87%	50%	44.44%	50.00%	51.85%	42.86%	34.38%	46.15%	69.70%	80.65%
	B27	FC	FC	FC	Mol. gen	Mol. gen	Mol. gen	Mol. gen	Mol. gen	Mol. gen	Mol. gen	Mol. gen	Mol. gen
		42.31%	56%	52%	50%	60.00%	66.67%	70.27%	66.67%	76.47%	68.42%	75.00%	76.92%
	CD	—	SSO	SSO	SSO	SSO	SSO	SSO	SSO	SSO	SSO	SSO	SSO
			66.67%	68.42%	68.18%	64.00%	73.33%	82.76%	65.71%	62.16%	72.97%	70.73%	71.05%
Antibodies	AHPA	—	—	—	—	—	—	—	—	Luminex	Luminex	Luminex	Luminex
										75.00%	80.00%	80.00%	100.00%
	ALO	Luminex (SA)	Luminex (SA)	Luminex (SA)	Luminex (SA)	Luminex (SA)	Luminex (SA)	Luminex (SA)	Luminex (SA)	Luminex (SA)	Luminex (SA)	Luminex (SA)	Luminex (SA)
		85.71%	88.89%	93.33%	93.33%	89.66%	93.10%	90.91	93.94	90.91	90.91	90.91	95
Serology	SER	PBMCs	PBMCs	PBMCs	PBMCs	PBMCs	PBMCs	PBMCs	PBMCs	PBMCs	PBMCs	PBMCs	PBMCs
		64.71%	68.42%	81.25%	81.25%	83.33%	80.00%	89.00%	86.00%	71.00%	71.00%	60.00%	75.00%
Crossmatch	XM	PBMCs	PBMCs	PBMCs	PBMCs	PBMCs	PBMCs	PBMCs	PBMCs	PBMCs	PBMCs	PBMCs	PBMCs
		73.91%	77.27%	76.19%	85%	72.73%	70.00%	71.43%	76.19%	61.90%	59.09%	54.55%	51.85%
	XMFC	—	Total lymph	Total lymph	Total lymph	Total lymph	Total lymph	Total lymph	Total lymph	Total lymph	Total lymph	Total lymph	Total lymph
			100%	100%	100%	100%	100%	100%	100%	92%	92%	100%	56%
Chimerism	CHM	—	—	STR	STR	STR	STR	STR	STR	STR and q/rt PCR	STR	STR	STR
				80.00%	42.86%	50.00%	60.00%	57.14%	53.85%	50%	57.14%	46.67%	53.33%

SSO, PCR-SSO (sequence-specific oligotyping); SSP, PCR-SSP (sequence-specific PCR); SBT, PCR-SBT (sequencing-based typing); NGS, PCR-NGS (next-generation sequencing); PBMCs, peripheral blood mononuclear cells; STR, short tandem repeats; q/rt, quantitative or real-time PCR.

The only scheme under 95% success rate (94.80%) was that of chimerism (Table 4), with most participants performing short tandem repeat (STR) analyses (Table 5) as the most performed method, followed by rtPCR alone or in combined with the STR analysis.

Within the interval: 95%–99% were serological typing, HLA-B27, HLA related to coeliac disease, chimerism, and crossmatches (Table 4). Serological typing was performed by most laboratories on peripheral blood mononuclear cells (PBMCs), while some others worked with T lymphocytes, which were magnetically isolated. Flow cytometry was the elected method for HLA-B27 until 2013, which was as frequent as molecular biology methods in 2014 (most frequently PCR-SSO, followed by rtPCR), and these were preferred from 2015 as shown in (Table 5).

All the low-resolution DNA, HLA, HPA, and KIR typing overall error rates (of 12 years) were under 1% together with anti-HLA and anti-HPA antibodies, and HLA-B27 and HLA-B*57:01 (Table 4). Up to 40.1% of KIR typing errors were due to allele variants (Supplementary Table S1). High-resolution HLA typing had a 1.1% error rate, with 60.1% of its errors attributable to improperly excluding null alleles (Supplementary Table S1). The poorest performance was that of chimerism (4.7%), where 62.2% errors were found in quantification and the second worst performance was of FC crossmatch, with a 4.22% error rate.

With regard to typing, homozygosity-reporting errors were common (Supplementary Table S1). Double writing of an allele should be used only when evidence of heterozygosity exists (EFI, 2019). Any other allele mismatch is here designated as random (wrong allele calls mostly), excluding ones originated by an improper or lacking null allele. All ambiguities that encompass a null allele must be resolved, wherever the polymorphism is located, unless it is evidenced that an expressed antigen is present on the cells (European Federation for Immunogenetics, 2019).

In the high-resolution HLA typing scheme, concordant results were accepted and therefore not penalized, regardless of whether allelic resolution was reached or not. The success rate in our series is similar to that reported by international next-generation sequencing–based workshops (Osoegawa et al., 2019)

**FIGURE 1:**
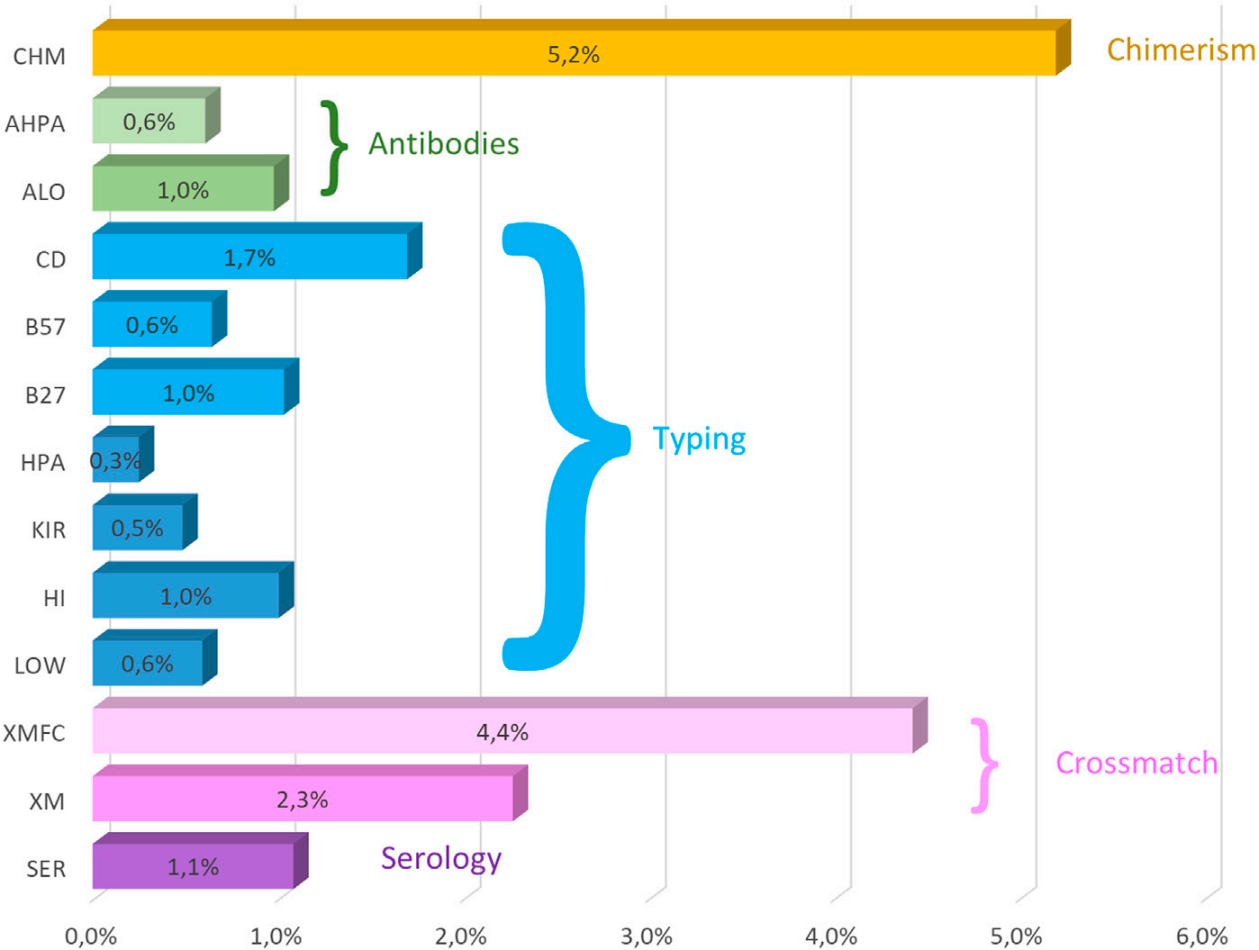
Mean (2011–2022) overall error rates of the GECLID EPT program. CHM, chimerism; AHPA, anti-HPA antibodies; ALO, anti-HLA antibodies; CD, coeliac disease–related HLA-B57, HLA-B*57:01 detection; HLA- B27, HLA-B27 status; HPA, HPA typing; KIR, KIR typing; HI, high-resolution DNA HLA typing; Low, low-resolution HLA DNA typing; XMFC, flow cytometry crossmatch; XM, CDC crossmatch; SER, HLA serological typing (O).

Crossmatches can be reported as whole results when no separation of cells is performed or as lymphocyte T (LT) or B (LB) separately. This separation was not implemented in CDC crossmatch until 2016. Crossmatch errors are very similar in both CDC and FC crossmatches (Figure 1).

In antibody schemes, both screening or detection and identification of specificities were evaluated by CDC and single antigen bead methods.

No differences in error rates could be evidenced when comparing different time periods but in the case of null alleles in high-resolution HLA DNA typing scheme from 2017 onward, when the steering committee decided not to admit generic annotations such as “every null allele excluded” and every excluded null allele had to be listed.

In 2022, 61.3% of the participating laboratories successfully passed every HLA EQA scheme they had taken, although 87.9% annual reports were satisfactory.

## 4 Discussion

Recent analyses of interlaboratory comparisons attribute a principal role to laboratory policies (Hicklin et al., 2023), HLA typing being the keystone for donor–recipient matching for both solid organ and hematopoietic stem cell transplantation. Many different laboratories perform HLA typing in the same country or region, and their results must meet the same standards so as to be reliable. Annual user meetings are held every single year where results are discussed and feedback, proposals, and complaints are received. A total of 12 user meetings were carried out from 2011 to 2022.

For all DNA-based EPT, the HLA typing results of the organizer are considered as correct results (European Federation for Immunogenetics, 2021). Our steering committee (integrated by five EFI-accredited laboratories) acts as the multicentric reference laboratory; however, due to the highly concordant results of participants to date, there is scarcely any need to consult them.

HLA-B*57:01 determination has high success rates in our EPT, in accordance with Meini et al. (2016), with a near absolute concordance irrespective of the method. HPA typing presented absolute concordance among participants as has been previously reported in other EPTs (Goldman et al., 2003). This is a low-complexity highly standardized test with minimum error rates, with therefore little improvement options regarding analytical performance.

Regarding high-resolution penalties, we should notice that null alleles are uncommon but not extremely rare, and they affect a significant number of unrelated donor searches regarding bone marrow transplantation (Smith et al., 2005). We occasionally observed unexpected results that were likely due to sample mix-ups, and pre-analytical or post-analytical issues rather than due to technical performance. Most typing penalties were due to nomenclature errors (null alleles not explicitly excluded) according to other EPTs reporting up to 12% nomenclature errors (Kekik Cinar et al., 2020). Automated analyses sometimes ended up in an incorrect HLA allele assignment. It is critical to review and validate HLA genotypes. It may be especially relevant regarding highly automated systems as the ones in the NGS HLA genotyping software (Meini et al., 2016). Error rates after making it mandatory to explicitly exclude any encompassed null allele indicate that this is the main source of error. Anyway, it is undistinguishable whether the laboratory did exclude the allele or not record it.

The HLA-B27 detection mean error is very similar to that found in a survey by the College of American Pathologists with molecular methods, even as 20%–25% of our participants reported using flow cytometry, which accounts for higher error rates (Peña et al., 2023). Some HLA-B27 alleles are now known to have a stronger association with the development of ankylosing spondylitis (AS), such as HLA-B*27:05, whereas HLA-B*27:06 and HLA-B*27:09 would not be related to AS risk. Perhaps high-resolution typing and risk assessment should be considered in the midterm.

Interpretation of HLA-DQ associated to coeliac disease and its risk were also a recurrent source of penalties. A key feature of coeliac disease is its strong dependence on the presence of susceptibility alleles encoding for HLA-DQ. Some EPT (Horan et al., 2018) revealed inconsistencies with current coeliac disease–reporting guidelines for genotype and clinical interpretation of the genotype data. Evidence-based guidelines are in this case not being consistently adhered to, in the same line as in our experience. Guidelines have been discussed in several of our meetings, even those specifically addressed to this topic, but adherence is not granted.

Regarding KIR typing, a former Spanish workshop (Planelles et al., 2016) found error rates slightly higher than the ones presented here. Success rates have improved over time from the beginning of the EPT as well, indicating that consolidated protocols and probably experienced interpretation account for better performances in this case. This fact may indicate that newer tests can specially benefit from the EPT results, allowing laboratories to improve their performance.

Whole blood crossmatches were as well somehow more prone to errors than LT or LB alone, possibly due to an overinterpretation of results (some participants alleged to have reported whole blood crossmatch positive when either LT or LB crossmatches were positive instead of performing it before lymphocyte separation). Although other EPTs (Putheti et al., 2022) report a decreasing number of laboratories performing CDC, the number of GECLID users of the scheme has grown in these 12 years. Some studies (Putheti et al., 2022) report a higher number of errors in LT than in LB in FC crossmatches, but we only found more LT errors in the first years of our EPT. This might point out that laboratory experience affects LT to a higher extent than LB. FC crossmatches may be too sensitive and lead to excessive wait times or to excessive excluding (Wrenn et al., 2018). Combined exercises of real and virtual crossmatches might help in establishing accurate cutoffs in order to improve efficiency.

The presence/absence of antibodies in each sample will be determined by consensus of at least 75% of the participating laboratories. When participants fail to report a specificity, 95% of the laboratories not reporting the specificity as positive are required to consider it as a negative consensus (European Federation for Immunogenetics, 2021). The specificities that are negative by omission between 75% and 95% of the participants will be included in the report as non-assessable negatives and will not be penalized. Most errors (89.7%) are due to identification of specificities, whereas screening of anti-class I/-class II antibodies accounts for 1.4%–1.9% errors and interpretation of anti-HLA-DQ or anti-HLA-DP from two-antigen coated beads would represent 5.4%. The overall mean error rate is 0.65% for anti-HLA and 0.60% regarding anti-HPA, indicating a good performance irrespective of the method or vendor (Israeli et al., 2015). The anti-HPA error rates in published workshops (Goldman et al., 2003; Bessos et al., 2005; Allen et al., 2013) are far higher than ours, but those exercises were performed on samples selected by their difficulties (differently to routine ones) and their assigned values were not that of consensus, and comparison to these EPTs is therefore useless.

Chimerism has the highest error rate in our EPT as some studies could not predict disease relapse by analyzing chimerism (Beck et al., 2006). This discrepancy could not only be caused by different patient populations or different underlying diseases but also by technical differences of the analysis. It is noticeable that this is also the only quantitative scheme in the HLA subprogram, and is therefore an added handicap. In fact, most penalties are due to deviant quantifications.

The common criteria for histocompatibility assays are the keystone for an efficient organ sharing system. The possibility of organ exchanges is closely linked to the importance of interlaboratory comparisons for histocompatibility and immunogenetics in order to build trustful networks. Most typing issues are due to nomenclature errors rather than technical errors. Manual validation by experienced immunologists is therefore a key point to get error-free results. Interpretation is anyway an error source regarding antibodies or risks, therefore has to be carefully performed only by qualified personnel. Recently incorporated tests obtain the highest profit of EPT participation. There is little heterogeneity of results of different assays used by participating laboratories, even when in-house assays are used. We have evidence enough to ascertain that HLA and immunogenetics assays in routine care in our area are highly reliable.

## Data Availability

The data analyzed in this study are not subject to any licenses/restrictions. Requests to access these data sets should be directed to responsable@geclid.es.
