# Supplementary material for: Analysis of immunogenetics interlaboratory comparisons’ success rates. External quality assurance system of the Spanish Society for Immunology GECLID-SEI
Source: Front Genet. 2024 Apr 30;15:1268728. doi: 10.3389/fgene.2024.1268728 (PMC11091402; doi:10.3389/fgene.2024.1268728)
Supplement: Supplementary file 1 [file Table1.DOCX]

Supplementary Table 1. Classification of errors within each scheme, mean error rates by scheme and percent error by type in the period and count of errors by year

|  | % error | **2011** | **2012** | **2013** | **2014** | **2015** | **2016** | **2017** | **2018** | **2019** | **2020** | **2021** | **2022** |
| --- | --- | --- | --- | --- | --- | --- | --- | --- | --- | --- | --- | --- | --- |
| Serological typing of HLA | 1.08% | 48 | 48 | 11 | 4 | 5 | 1 | 2 | 3 | 4 | 4 | 2 | 0 |
| Homozygous | 56.8% | 28 | 32 | 8 |  | 2 |  |  |  | 1 | 4 |  |  |
| Random | 43.2% | 20 | 16 | 3 | 4 | 3 | 1 | 2 | 3 | 3 |  | 2 |  |
| HLA-B27 | 1.03% | 1 | 1 | 8 | 2 | 3 | 2 | 5 | 6 | 1 | 4 | 6 | 1 |
| HLA*B57:01 | 0.64% |  | 2 | 1 | 1 |  | 16 | 0 | 9 | 8 | 1 | 8 | 3 |
| Random | 57.1% |  | 2 |  |  |  | 12 |  | 4 | 4 |  | 4 | 2 |
| Screening | 40.8% |  |  |  | 1 |  | 4 |  | 5 | 4 | 1 | 4 | 1 |
| Coeliac disease related HLA | 1.70% |  | 11 | 4 | 5 | 5 | 43 | 12 | 26 | 27 | 20 | 27 | 7 |
| Random | 58.3% |  |  | 7 | 4 |  | 4 | 32 | 8 | 12 | 20 | 12 | 10 |
| Risk | 38.0% |  |  | 4 |  | 5 | 1 | 11 | 4 | 14 | 7 | 8 | 17 |
| Cytotoxicity Crossmatch* | 2.27% | 75 | 33 | 30 | 27 | 30 | 54 | 19 | 25 | 56 | 16 | 62 | 34 |
| Whole | 58.5% | 75 | 33 | 29 | 27 | 30 | 54 | 18 | 22 | 25 | 11 | 32 | 16 |
| LB | 21.7% |  |  |  |  |  |  | 1 |  | 15 | 3 | 17 | 10 |
| LT | 19.8% |  |  |  |  |  |  |  | 3 | 16 | 2 | 13 | 8 |
| Flow Cytometry Crossmatch | 4.43% | 30 | 36 | 21 | 41 | 53 | 54 | 68 | 66 | 45 | 73 | 40 |  |
| LB | 50.9% | 11 | 15 | 5 | 21 | 26 | 38 | 41 | 34 | 23 | 34 | 20 |  |
| LT | 49.0% | 19 | 20 | 16 | 20 | 27 | 16 | 27 | 32 | 22 | 39 | 20 |  |
| Anti-HLA antibodies | 0.98% | 336 | 961 | 596 | 868 | 1104 | 948 | 579 | 1174 | 902 | 812 | 524 | 646 |
| Screening (beads) | 1.9% | 6 | 16 | 7 | 8 | 8 | 7 | 38 | 25 | 18 | 16 | 15 | 17 |
| SA clases | 1.5% | 6 | 19 | 8 | 9 |  | 7 | 12 | 20 | 12 | 17 | 18 | 17 |
| SA specificities | 89.7% | 277 | 811 | 501 | 710 | 1010 | 862 | 525 | 1125 | 845 | 760 | 469 | 580 |
| SA interpretacion | 5.4% |  | 55 | 59 | 132 | 78 | 59 | 4 | 29 | 27 | 19 | 21 | 31 |
| screning CDC | 0.4% | 11 |  | 13 | 9 | 2 | 3 |  | 2 |  |  | 1 |  |
| specificities CDC | 1.6% | 36 |  |  |  | 6 | 110 |  | 2 |  |  |  | 1 |
| Low resolution HLA DNA typing | 0.59% | 33 | 36 | 36 | 17 | 29 | 28 | 55 | 12 | 16 | 34 | 11 | 8 |
| Homozygous | 27.0% | 10 | 15 | 14 | 5 |  | 10 | 15 | 4 | 4 |  | 3 | 5 |
| Random | 53.7% | 23 | 21 | 22 | 14 |  | 18 | 40 | 8 | 12 |  | 8 | 3 |
| High resolution HLA DNA typing | 1.01% | 30 | 33 | 30 | 18 | 25 | 16 | 81 | 59 | 58 | 75 | 96 | 101 |
| Homozygous | 13.3% | 3 | 14 | 0 | 0 | 9 | 1 | 7 | 3 | 2 | 10 | 12 | 22 |
| Random | 26.5% | 27 | 4 | 21 | 8 | 9 | 6 | 28 | 23 | 7 | 9 | 5 | 18 |
| null | 60.1% | 1 | 15 | 9 | 10 | 6 | 9 | 46 | 33 | 49 | 56 | 79 | 61 |
| Chimerism | 5.2% |  |  | 13 | 12 | 5 | 8 | 10 | 14 | 20 | 10 | 12 | 7 |
| Random | 42.3% |  |  | 10 | 7 | 1 | 6 | 3 | 8 | 6 | 2 | 3 | 1 |
| Quantification | 62.2% |  |  | 3 | 5 | 9 | 2 | 7 | 6 | 14 | 8 | 9 | 6 |
| KIR Typing | 0.49% |  |  |  |  |  | 26 | 24 | 24 | 14 | 10 | 10 | 6 |
| Random | 63.2% |  |  |  |  |  | 23 | 21 | 12 | 2 | 7 | 2 | 5 |
| Variants | 40.4% |  |  |  |  |  | 3 | 7 | 12 | 12 | 3 | 8 | 1 |
| HPA typing | 0.25% |  |  |  |  |  |  | 0 | 0 | 6 | 3 | 0 | 0 |
| Anti HPA antibodies | 0.61% |  |  |  |  |  |  |  | 0 | 0 | 3 | 1 | 0 |

Homozygous: notation errors misleading on homozygosity of a locus; random: alleles or values not concordant with assigned value, excluding those included elsewhere; Risk: risk evaluation not correct; whole: performed without separation of T and B lymphocytes; LT: performed on isolated T lymphocytes; LB: performed on isolated B lymphocytes; SA classes: evaluation of ant HLA class I or II with SA beads; null: not excluding null alleles; variants: wrong assignment of variants of KIR alleles
